# An *in vitro* experimental pipeline to characterize the epitope of a SARS-CoV-2 neutralizing antibody

**DOI:** 10.1128/mbio.02477-23

**Published:** 2023-12-06

**Authors:** Kristina E. Atanasoff, Luca Brambilla, Daniel C. Adelsberg, Shreyas Kowdle, Christian S. Stevens, Stefan Slamanig, Chuan-Tien Hung, Yanwen Fu, Reyna Lim, Linh Tran, Robert Allen, Weina Sun, J. Andrew Duty, Goran Bajic, Benhur Lee, Domenico Tortorella

**Affiliations:** 1 Department of Microbiology, Icahn School of Medicine at Mount Sinai, New York, New York, USA; 2 Graduate School of Biomedical Sciences, Icahn School of Medicine at Mount Sinai, New York, New York, USA; 3 Swammerdam Institute for Life Sciences, University of Amsterdam, Amsterdam, The Netherlands; 4 Sorrento Therapeutics, Inc., San Diego, California, USA; 5 Center for Therapeutic Antibody Development, Icahn School of Medicine at Mount Sinai, New York, New York, USA; Washington University in St Louis School of Medicine, St. Louis, Missouri, USA

**Keywords:** SARS-CoV-2, neutralizing antibody, viral resistance, epitope mapping, structural analysis, cryo-EM

## Abstract

The coronavirus disease 2019 (COVID-19) pandemic caused by severe acute respiratory syndrome-coronavirus-2 (SARS-CoV-2) has led to over 770 million cases and >6.9 million deaths worldwide. We identified a panel of human neutralizing monoclonal antibodies (mAbs) targeting the SARS-CoV-2 Spike protein using Harbour H2L2 transgenic mice immunized with Spike receptor-binding domain (RBD) (J. A. Duty, T. Kraus, H. Zhou, Y. Zhang, et al., Med 3:705–721, 2022, https://doi.org/10.1016/j.medj.2022.08.002). Representative antibodies from genetically distinct families were evaluated for the inhibition of replication-competent VSV expressing SARS-CoV-2 Spike (rcVSV-S) in the place of VSV-G. One mAb clone denoted FG-10A3 and its therapeutically modified version STI-9167 effectively inhibited infection and *in vivo* proliferation of early variants of SARS-CoV-2 including Omicron BA.1 and BA.2 and corresponding pseudoviruses and rcVSV-S variants (Duty et al.). To define the epitope of the broadly reactive FG-10A3 mAb, we generated mAb-resistant rcVSV-S virions and performed structural analysis of the antibody/antigen complex using cryo-electron microscopy (EM). FG-10A3/STI-9167 is a Class 1 antibody that prevents Spike-ACE2 binding by engaging a region within the Spike receptor binding motif. Sequencing of mAb-resistant rcVSV-S virions identified F486 as a critical residue for mAb neutralization, with structural analysis revealing that both the variable heavy and light chains of STI-9167 bound the disulfide-stabilized 470–490 loop at the Spike RBD tip. Furthermore, neutralization studies using rcVSV-S F486 point mutants and currently-circulating variants Omicron BA.5, XBB.1.5, and BQ.1.1 that contain a V or P at position 486 further supported the model in which residue 486 is an important residue for FG-10A3 inhibition. This work provides an experimental strategy to define the neutralizing capacity and limitations of mAb therapeutics against emerging SARS-CoV-2 variants.

**IMPORTANCE:**

The COVID-19 pandemic remains a significant public health concern for the global population; the development and characterization of therapeutics, especially ones that are broadly effective, will continue to be essential as severe acute respiratory syndrome-coronavirus-2 (SARS-CoV-2) variants emerge. Neutralizing monoclonal antibodies remain an effective therapeutic strategy to prevent virus infection and spread so long as they recognize and interact with circulating variants. The epitope and binding specificity of a neutralizing anti-SARS-CoV-2 Spike receptor-binding domain antibody clone against many SARS-CoV-2 variants of concern were characterized by generating antibody-resistant virions coupled with cryo-EM structural analysis and VSV-spike neutralization studies. This workflow can serve to predict the efficacy of antibody therapeutics against emerging variants and inform the design of therapeutics and vaccines.

## INTRODUCTION

The coronavirus disease 2019 (COVID-19) pandemic caused by severe acute respiratory syndrome-coronavirus-2 (SARS-CoV-2) is an unprecedented global public health challenge, with over 770 million cases and >6.9 million deaths worldwide as of August 2023 (1). In the last 18 years, bat coronaviruses SARS-CoV (2003), Middle East respiratory syndrome coronavirus (2012), and SARS-CoV-2 (2019) have jumped from animal reservoirs to cause significant human outbreaks. The entry of SARS-CoV and SARS-CoV-2, as well as many other bat CoVs, is mediated via engagement of the envelope spike glycoprotein (denoted “Spike” or S) with the human angiotensin-converting enzyme 2 (ACE2) cell surface protein (2–4). This is followed by activation and cleavage of Spike mediated by host cell proteases, primarily either transmembrane protease, serine 2 (TMPRSS2), which permits virus-host membrane fusion at the cell surface, or cathepsin proteases, whose activity occurs during clathrin-mediated endocytosis of virions (5). SARS-CoV-2 Spike is a type I membrane glycoprotein comprising an S1 and S2 domain that binds to ACE2 via the receptor-binding domain (RBD) contained within S1. Monoclonal antibodies (mAbs) directed to the RBD of SARS-CoV-2 demonstrated efficacy in limiting disease symptoms and hospital stay (6–11). However, due to the rise of new variants of concern (VoCs), the FDA’s Emergency Use Authorization of mAb therapeutics targeting Spike, such as LYCoV1404 (bebtelovimab) and tixagevimab/cilgavimab (Evusheld), has been revoked due to demonstrated limited efficacy *in vitro* against newly arisen VoCs (12). This combined with predicted corresponding neutralizing serum titers in clinical trials indicated that these mAb therapeutics no longer met the FDA’s threshold as effective clinical treatments. Despite these findings, neutralizing antibodies remain a promising therapeutic strategy to prevent virus infection and spread.

SARS-CoV-2 neutralizing mAbs target multiple regions of Spike by blocking virus binding to ACE2 or subsequent fusion with the host cell membrane. Such mAbs can target either the S1 or S2 domain of Spike, with the most potent being those against the RBD within the S1 domain (13). mAbs can be classified as binding to S1 (recognizing S1’s RBD or N-terminal domain) or S2 (recognizing the stem helix or fusion peptide). The anti-S1 mAbs that interact with Spike RBD can be further subdivided into four classes: Class 1 and Class 2 mAbs, which both overlap with the receptor-binding motif (RBM) and are distinguished by their ability to recognize RBD in exclusively the “up” conformation of Spike (Class 1) or in both “up” and “down” conformations (Class 2); Class 3 mAbs, which interact with the RBD outside of the RBM independent of Spike conformation; and Class 4 mAbs, which recognize conserved regions within the RBD that do not directly block ACE2 binding. These mAbs represent biologics whose neutralization capacity may diminish as new VoCs arise. However, mAbs that target conserved regions of Spike essential for virus fitness would likely maintain their therapeutic efficacy against emerging VoCs.

We generated a panel of neutralizing mAbs that inhibit SARS-CoV-2 infection from Harbour H2L2 mice that are transgenically modified to encode human immunoglobulin variable regions and rodent constant regions (14, 15). Of the identified mAbs, one neutralizing mAb, designated Family G (FG)-10A3 or its therapeutically modified version STI-9167, had broad-spectrum neutralization activity against the early circulating SARS-CoV-2 VoCs, including Omicron (BA.1 and BA.2), in both pseudotyped and live virus infectivity assays (14). To define the epitope of the broadly reactive mAb FG-10A3/STI-9167, we generated FG-10A3 mAb-resistant virions by utilizing replication-competent (rc) VSV expressing the SARS-CoV-2 WT-D614G (WA-1) Spike protein and defined the structure of STI-9167 in complex with SARS-CoV-2 Spike (WA-1). Our study identified the phenylalanine 486 residue on the Spike protein as an important residue for FG-10A3/STI-9167’s epitope recognition enabling the mAb’s neutralizing activity against SARS-CoV-2 VoCs; while establishing a robust *in vitro* experimental strategy to define the neutralizing capacity of mAb therapeutics.

## MATERIALS AND METHODS

### Cells and antibodies

Vero E6 cells (ATCC #CCL-81) and Vero cells expressing ACE2 and transmembrane protease, serine 2 (TMPRSS2) (Vero-ACE2/TMPRSS2) (16) were maintained in Dulbecco’s modified Eagle’s medium (DMEM, Corning, 10-013-CV) supplemented with 10% heat-inactivated fetal bovine serum (FBS, ThermoFisher Scientific), 1 mM HEPES (Corning, 25-060-CI), 100 U/mL penicillin, and 100 g/mL streptomycin (100× Pen/Strep, Corning, 30-002-CI). Cell lines were cultured at 37°C with 5% CO_2_. Human monoclonal anti-SARS-CoV-2 Spike antibodies (mAbs) were generated, sequenced, and humanized as described previously (14). The antibodies were purified from the supernatant of 293ExpiF cells transfected with the respective heavy and light chains of each clone. The FG-10A3 clone (heavy chain CDR3: QVQLVESGGGVVQPGRSLRLSCAASGFTFSSYGMNWVRQAPGKGLEWVAIIWYDGNNTYYVDSVKGRFTISRDNSKNTLYLQMNSLRAEDTAVYYCARKDGSKTYYGYYFDYWGQGTLVTVSS; light chain CDR3: DIQMTQSPSSLSASVGDRVTITCRASQSIHSFLNWYQQKPGKPPNLLIYAASSLQSGLPSRFSGSGSGTDFTLTISSLQPEDFATYYCQQSYITPPTFGHGTKVEIK) was modified with a LALA sequence to prevent ADE activity to generate the mAb STI-9167 (STI-9167 [10A3YQYK] HC CDR123 AA sequence by kabat: QVQLVESGGGVVQPGRSLRLSCAASGFTFSSYGMNWVRQAPGKGLEWVAIIWYYGNNKYYVDSVKGRFTISRDNSKNTLYLQMNSLRAEDTAVYYCARKDYSKTYYGYYFDYWGQGTLVTVSS; STI-9167 10A3YQYK LC CDR123 AA sequence by kabat: DIQMTQSPSSLSASVGDRVTITCRASQSIHSFLNWYQQKPGKPPNLLIYAASSLQSGLPSRFSGSGSGTDFTLTISSLQPEDFATYYCQQSYITPPTFGQGTKVEIK). Underlined residues represent modifications between the respective amino acid sequences.

### Sequencing of viral RNA

Viral RNA was extracted using the QIAamp Viral RNA Mini Kit (Qiagen). The Spike region of VSV-S virions was amplified via the SuperScript IV One-Step RT-PCR System (Thermo Fisher Scientific) and purified using the SPRIselect-PCR Purification and Cleanup Kit (Beckman Coulter Life Sciences). Libraries were prepared using the Illumina DNA Prep Tagmentation Kit and barcoded using the Nextera DNA CD Indexes (Illumina). One hundred fifty base pair paired-end sequencing was performed on an Illumina iSeq 100.

### Sequencing analysis

For the bioinformatic analysis of raw sequencing data, an in-house analysis pipeline was used to process raw FASTQ files and identify all variants called at a threshold of 1%. Variants shown meet a threshold of 10% of all reads in any one sample at any point during passaging, result in an amino acid change, and are found at positions with a read depth of at least 5,000 reads. First, reads were processed and mapped using SAMtools (17) and BWA-MEM (18) against SARS-CoV-2-Spike (WA-1) (19). Bcftools mpileup (18) and bedtools genomecov (20) were then used to identify all variants and their relative frequency across samples. Further analysis was performed in R using the tidyR (21) and Biostrings (22) packages.

### Generation of VSV-eGFP-CoV-2 Spike (Δ21 aa), point mutants, and helper plasmids

We cloned the genomic sequences of all VSV-S pseudoviruses and helper plasmids into expression vectors as previously described (18). Briefly, we replaced the VSV-G open reading frame of a pEMC vector expressing VSV-eGFP with SARS-CoV2-S WT-D614G (WA-1) or specific variants B.135 (Beta), B.617.2 (Delta), B.1.1.529 (Omicron BA.1), and Omicron subvariants BA.5, BQ.1.1, and XBB.1.5, all of which were truncated and thus lacking the final 21 amino acids (23). The sequences of VSV-S(WA-1) and VSV-S(Beta) are available at GenBank (accession numbers: MW816497 and MW816499). VSV-S was adapted to the new Omicron variants BA.5, BQ.1.1, and XBB.1.5 by introducing the appropriate variant-specific mutations into the WA-1 backbone *in silico* (including F486V for BA.5 and BQ.1.1 and F486P for XBB.1.5, respectively). The newly designed Spikes were obtained as gBlock gene fragments from Integrated DNA Technologies and cloned into the VSV plasmid. We generated point mutations at the F486 residue by primer-mediated site-directed mutagenesis of the parental (WA-1) Spike. Forward and reverse primers were designed to mutate phenylalanine at position 486 to serine (F486S), leucine (F486L), or valine (F486V), and generate a 20-bp overlap between fragments. Point-mutant Spike fragments were cloned into the MluI_PacI digested VSV-eGFP backbone via InFusion seamless cloning (TakaraBio). The sequences of VSV N, P, M, and L were cloned into pCI vectors to generate helper plasmids for viral rescue. These accessory plasmids were a generous gift from Dr. Benjamin tenOever (NYU School of Medicine).

### VSV-eGFP-CoV-2 rescue from cDNA

For all VSV pseudoviruses, 3 × 10^5^ BHK-ACE2 cells per well were seeded onto 6-well plates. The next day, 2,000 ng of pEMC-VSV-eGFP-CoV-2 Spike, 2,500 ng of pCAGGS-T7opt (24), 850 ng of pCI-VSV-N, 400 ng of pCI-VSV-P, and 100 ng of pCI-VSV-L were mixed with 5.5 µL of Plus reagent and 8.9 µL of Lipofectamine LTX (Invitrogen) in 200 µL of OptiMEM medium (Gibco). Thirty minutes later, the transfection mixture was added dropwise onto plated BHK-ACE2 cells. The medium was replaced 24 h post-transfection with DMEM supplemented with 10% FBS, and cells were monitored daily for fluorescence with a Celigo imaging cytometer (Nexcelom Bioscience). Three to five days post-transfection, cells exhibited extensive GFP-positive syncytia and cell-free viral spread to previously uninfected cells. The supernatant containing pseudovirus was collected, clarified by centrifugation for 5 minutes at 400 g, and amplified on Vero-CCL81-TMPRSS2 cells (16). The rcVSV-S virions were titered on Vero-ACE2/TMPRSS2 cells based on the number of GFP positive cells/mL.

### VSV-S neutralization assays

**Fig 1:**
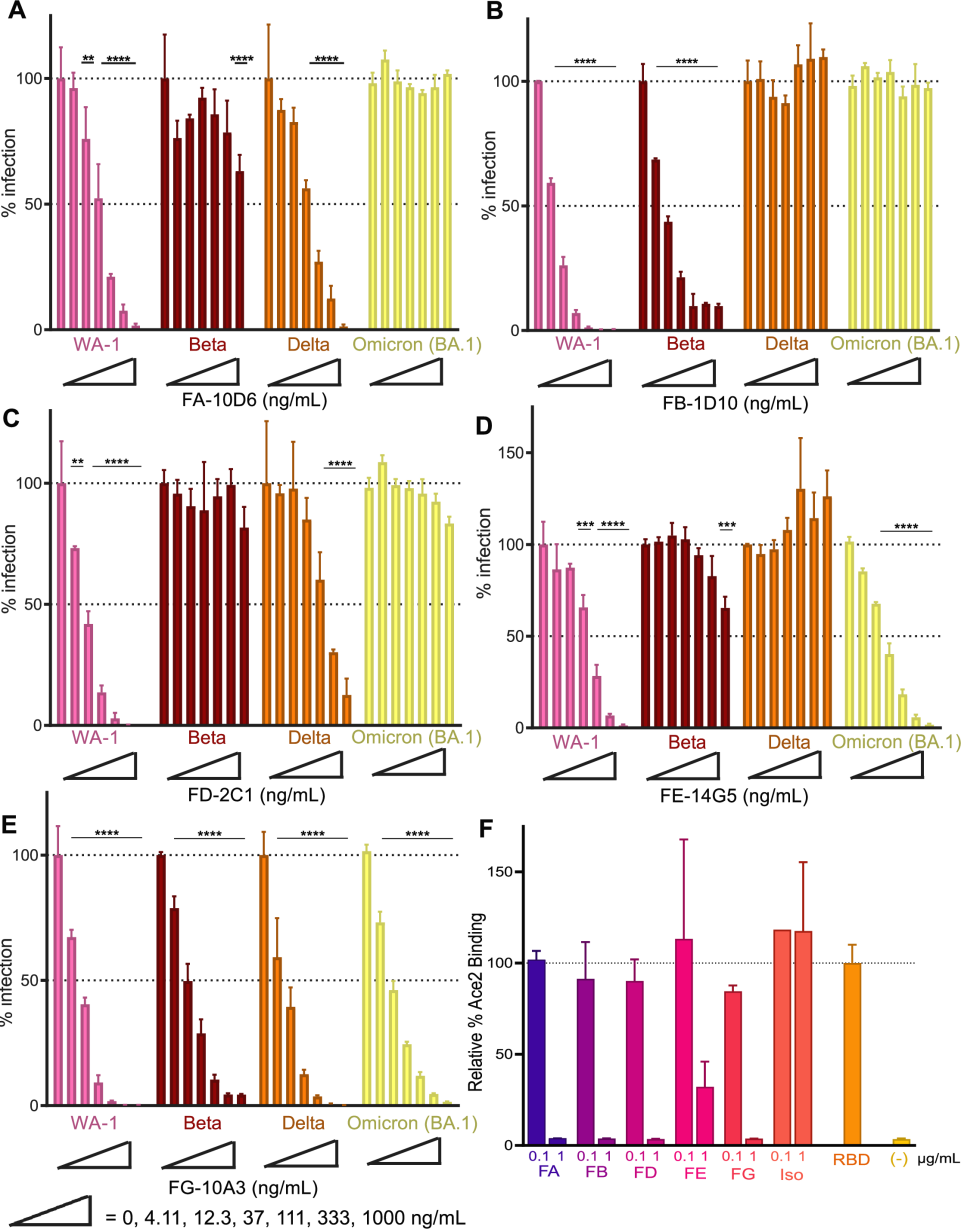
Analysis of anti-SARS-CoV-2 mAbs against rcVSV-S variants. FA-10D6 (**A**), FB-1D10 (**B**), FD-2C1 (**C**), FE-14G5 (**D**), and FG-10A3 (**E**) mAbs were evaluated with a neutralization assay against rcVSV-S WA-1 (WA-1) and rcVSV-S variants B.135 (Beta), B.617.2 (Delta), and B.1.1.529 (Omicron BA.1). RcVSV-S virions (MOI = 0.2) were preincubated with increasing concentrations of mAbs (0–1,000 ng/mL) followed by infection of Vero-ACE2/TMPRSS2 cells and analyzed using the neutralization assay. Error bars represent the standard deviation from the mean of three samples (serving as technical replicates). Data depicted are that of a single representative experiment out of two independent experimental replicates performed. Statistical significance is denoted as follows: **P < 0.01; ***P < 0.001; and ****P < 0.0001. (**F**) Recombinant SARS-CoV-2 (WA-1) RBD-Fc was preincubated with 0.1 and 1 µg/mL of FA-10D6, FB-1D10, FD-2C1, FE-14GE, or FG-10A3 and then added to HEK-293 cells expressing hACE2 followed by flow cytometry analysis. Percent ACE2 binding was determined relative to binding of untreated RBD-Fc to ACE2-expressing cells. Error bars represent the standard deviation from the mean of duplicate samples.

**Fig 2:**
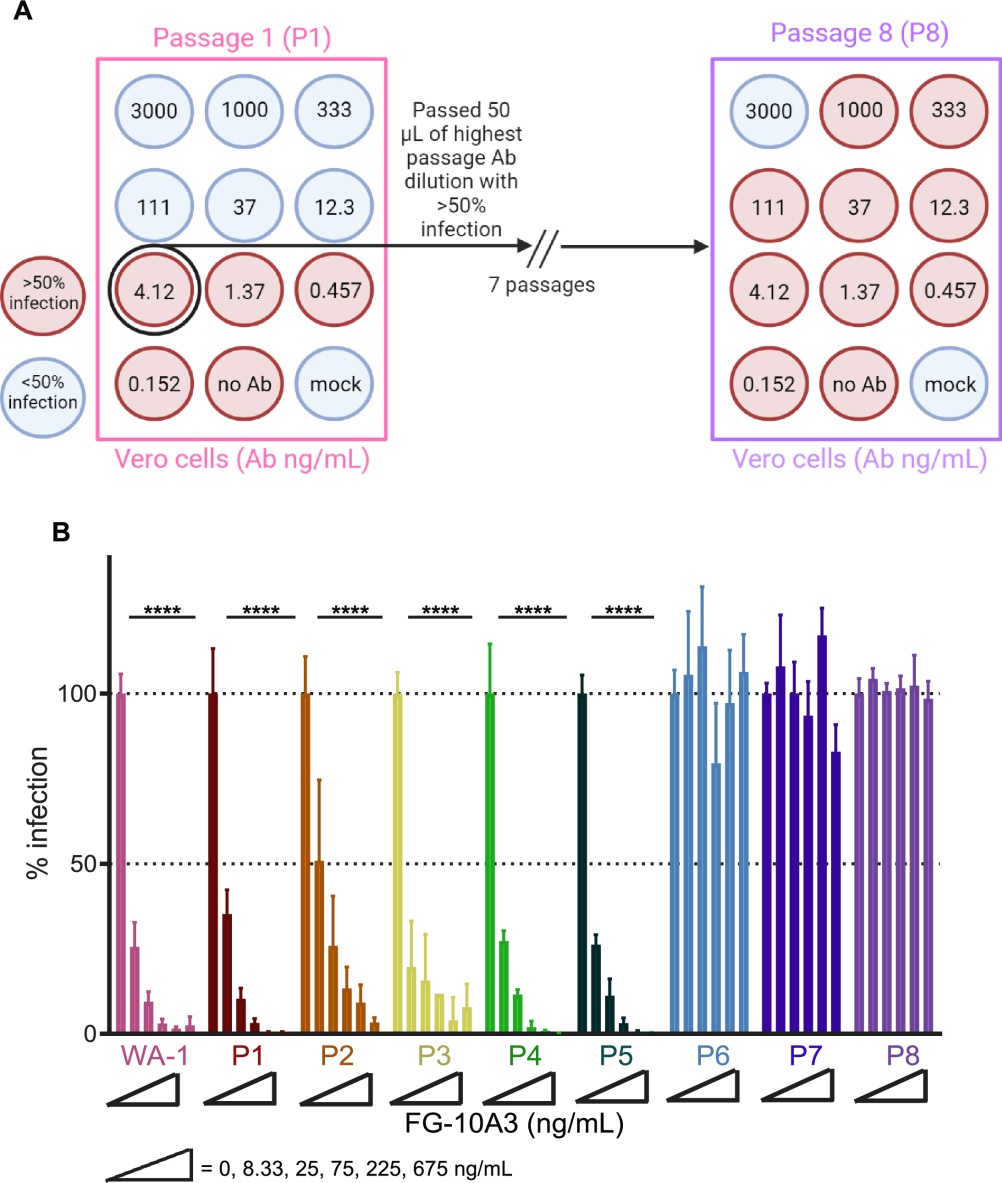
Generation of rcVSV-S WA-1 virions resistant to FG-10A3. (**A**) Schematic diagram illustrating our strategy for generating FG-10A3 mAb-resistant rcVSV-S WA-1 virions. Vero-E6 cells infected with rcVSV-S WA-1 (MOI = 0.1) in the presence of FG-10A3 (0–3,000 ng/mL) were analyzed using the neutralization assay. Supernatant from the wells with >50% infection treated with the highest concentration of FG-10A3 was considered passage 1 (P1) and selected for subsequent rounds of incubation with FG-10A3 (0–3,000 ng/mL). Using this strategy, rcVSV-S WA-1 was passaged for a total of eight passages, denoted P1–P8. (**B**) FG-10A3-incubated rcVSV-S from P1–P8 was assessed using an FG-10A3-based neutralization assay with infection determined relative to untreated rcVSV-S WA-1. Error bars represent the standard deviation from the mean of three samples. Statistical significance is denoted as follows: ****P < 0.0001.

Vero-ACE2/TMPRSS2 cells (1 × 10^4^/well) were plated in a 96-well plate and placed at 37°C/5% CO^2^ overnight (~18 h). The cells were infected with rcVSV-S virions (MOI = 0.2 in 50 µL DMEM) pre-incubated with anti-Spike mAbs (0, 4.1, 12.3, 37, 111, 333, and 1,000 ng/mL [Fig. 1]; 0, 8.3, 25, 75, 225, and 675 ng/mL [Fig. 2; see Fig. 4]; 0, 0.625, 1.25, 2.5, 5, and 10 µg/mL [see Fig. 6]; 0, 0.12, 0.37, 1.11, 3.33, 8.33, 25, 75, 225, and 675 ng/mL [Fig. S1]) at 4°C for 1 h. The total 100 µL of virus mixed with mAbs was then added to cells, followed by incubation for ~18 hpi at 37°C. Cells were fixed in 4% paraformaldehyde, permeabilized (0.3% Triton X-100 [ThermoFisher, HFH10] in PBS), and then stained with Hoechst reagent (0.01 µg/mL in PBS) to quantify total cells. Virus neutralization was quantified using a plate-based imaging Celigo cytometer (Nexelcom Bioscience, Version 4.1.3.0) based on the number of GFP-positive cells as well as the total number of cells per well. Relative percent infection was determined using the number of GFP-positive cells in wells infected, with rcVSV-S alone as 100% infection.

### Generation of mAb-resistant VSV-S(WA-1) virions

Vero E6 cells (2.5 × 10^4^/well) were plated in a 24-well plate and placed at 37°C/5% CO_2_ overnight (~18 h). The cells were infected with rcVSV-S (WA-1) (MOI = 0.1 in 500 µL DMEM) pre-incubated with anti-Spike mAb FG-10A3 (0, 0.15, 0.46, 1.4, 4.1, 12.3, 37, 111, 333, 1,000, and 3,000 ng/mL final concentrations in 1 mL DMEM) at 4°C for 1 h. The total 1 mL of virus mixed with mAbs was added to cells, followed by incubation at 37°C. At 72 h post-infection, the percentage of GFP-positive cells was determined using a Celigo cytometer, and the supernatant from the wells with >50% of infection (50 µL) was selected for incubation with FG-10A3 (0–3,000 ng/mL). Passaged virus was defined as “resistant” to FG-10A3 when >600 ng/mL of FG-10A3 (100 × EC_50_ [6 ng/mL]) was unable to achieve >50% neutralization of infection. Once resistance was elicited, the virus was passaged twice more with FG-10A3 (0–3,000 ng/mL) to verify that viral resistance could be maintained, yielding a total of eight viral passages. Stocks of these eight passages were grown and amplified in the absence of mAb, then titered on Vero-ACE2/TMPRSS2 cells prior to the assessment of mAb-mediated neutralization. To control for amino acid changes arising through rcVSV-S passaging, rcVSV-S WA-1 incubated with 1,000 ng/mL of an isotype control was also collected over eight passages.

### hACE2/antibody competition assay

Purified monoclonal antibodies (0.1 and 1 µg/mL) were pre-incubated with Wuhan Spike RBD-mG2a Fc fusion protein (0.25 mg/mL) for 30 minutes on ice in FACS buffer (1× PBS, 0.5% BSA, and 2 mM EDTA) followed by addition to HEK-293 cells expressing human ACE2 for 30 minutes on ice. Cells were washed 2× with FACS buffer and resuspended in FACS buffer containing goat anti-mouse IgG-APC secondary (1:1,000). Cells were incubated for 30 minutes on ice, washed 2× with FACS buffer, and resuspended in FACS buffer for analysis on an Intellicyte HFTC (in duplicate). Mean fluorescent intensities were determined and normalized using RBD as 100% binding. Secondary alone was included as a negative control.

### *K*_*D*_ determination

Biolayer interferometry assays were performed using the Octet RED 96 instrument (SartoriusAG) to determine the association rates (*k*_on_), dissociation rates (*k*_dis_), and affinity (*K*_*D*_) for the antibody. Purified Spike RBD-mouse Fc fusion protein was loaded onto anti-mouse Fc IgG capture (AMC) biosensors using a constant 5 µg/mL concentration for 10 minutes at 20°C. To determine the *k*_on_, the sensors were exposed to antibodies starting at a concentration of 1 µg/mL (twofold dilutions [1–0.0078 µg/mL in PBS] for 3 minutes). To determine *k*_dis_ values, dissociation was measured over the course of 3 minutes while the sensors were in PBS buffer. *K*_*D*_ values were calculated as a ratio of *k*_dis_/*k*_on_. A binding model of 1:1 resulted in the best fit for each antibody, and the resulting *R*^2^ values are given for each antibody in Table 1.

**TABLE 1:** SARS-CoV-2 WA-1 Spike RBD binding affinity for mAbs

Antibody	CDR3 (H)	*K*_*D*_^*a*^ (M)
FA-10D6	12 aa	5.74 E−11
FB-1D10	19 aa	5.63 E−11
FD-2C1	14 aa	3.71 E−10
FE-14G5	16 aa	1.12 E−10
FG-10A3	16 aa	1.25 E−10

^*a*^ Determined using RBD.

### Cryo-EM sample preparation and data collection

SARS-CoV-2 HexaPro Spike (25) was incubated with STI-9167 Fab at 2.5 mg/mL and a molar ratio of 1.5:1 Fab:Spike for 20 minutes at 4°C. Immediately before grid preparation, fluorinated octyl maltoside was added to the pre-formed complex at 0.02%, wt/vol final concentration. 3 µL aliquots were applied to UltrAuFoil gold R0.6/1 grids and subsequently blotted for 6 seconds at blot force 1, then plunge‐frozen in liquid ethane using an FEI Vitrobot Mark IV. Grids were imaged on a Titan Krios microscope operated at 300 kV and equipped with a Gatan K3 Summit direct detector. A total of 20,590 movies were collected in counting mode at 16e−/pix/second at a magnification of 105,000, corresponding to a calibrated pixel size of 0.826 Å. Defocus values were from −0.5 to −1.5 µm.

### Cryo-EM data processing

Movies were aligned and dose-weighted using MotionCorr2 (26). Contrast transfer function estimation was done in cryoSPARC v3.3.1 using Patch CTF, and particles were picked with cryoSPARC’s blob picker. The picked particles were extracted with a box size of 512 pixels, with 4× binning, and subjected to a 2D classification. Selected particles were then subjected to a second round of 2D classification. An initial model was generated on the 1,410,814 selected particles at 6 Å/pixel with four classes. The best class, containing 558,457 particles, was selected for further processing. After one round of non-uniform refinement, without imposed symmetry, the particles were subjected to 3D classification with five classes. Of these, the best three classes, containing 321,918 particles in total, were combined, re-extracted without binning with a box size of 512 pixels, and selected for further rounds of non-uniform refinement with local CTF refinement, yielding the final global map at a nominal resolution of 2.53 Å. The protomer with the best Fab volume was subjected to local refinement with a soft mask extended by six pixels and padded by 12 pixels encompassing the RBD and Fab. A second round of local refinement was performed with a soft mask encompassing the RBD and variable domains of the Fab. This yielded the final local map at 3.16 Å resolution. The two half-maps from the global or local refinement were used for sharpening in DeepEMhancer (27). The reported resolutions are based on the gold-standard Fourier shell correlation of 0.143 criterion.

### Model building and refinement

RBD from Protein DataBank (ID: 6M0J) and AlphaFold2-predicted Fab variable domains were fit into focus-refined maps using UCSF ChimeraX (28) and then manually built using COOT (29). N-linked glycans were built manually in COOT using the glyco extension and their stereochemistry and fit to the map were validated with Privateer (30). The model was then refined in Phenix (31) using real-space refinement and validated with MolProbity (32).

### Statistics and reproducibility

All statistical tests were performed using GraphPad Prism 9 software (La Jolla, CA, USA). The half-maximal effective concentration (EC_50_) values for each anti-Spike mAb were calculated using three-parameter non-linear regression analysis after the mAb concentrations were transformed to log scale. Significance and adjusted *P*-values for mAbs’ inhibitory effects on infection were calculated via two-way ANOVA statistical tests, where the mean relative percent infection of each experimental condition was compared to the mean relative percent infection of cells treated with virus alone. For all figures, error bars represent the standard deviation of the mean. Sample size and replicates for each experiment are indicated in the figure legends. Technical replicates were prepared in parallel within one experiment, and experimental replicates were performed on separate days.

## RESULTS

### Human anti-RBD neutralizing mAbs block infection of rcVSV-S variants

A panel of human neutralizing mAbs against SARS-CoV-2 (Wuhan) was identified from Harbour H2L2 mice immunized with a SARS-CoV-2 Spike receptor-binding domain (Wuhan)-mouse Fc fusion protein (14). Hybridoma clones demonstrating: (i) specific binding to SARS-CoV-2 Spike (Wuhan) expressed on 293ExpiF cells; (ii) RBD binding as determined by ELISA; and (iii) replication-competent VSV-S (WA-1) neutralization of >50% were selected for nucleotide sequence analysis to identify unique clones, as described by Duty et al. (14). The neutralizing clones were classified into antibody “families” (Family A, B, D, E, F, and G) based on amino acid sequence, V(D)J gene usage, and complementarity-determining region 3 (CDR3) junctions ranging in length from 12 to 19 aa. These initial studies defined Family G clone 10A3 (FG-10A3) as a broadly neutralizing mAb with therapeutic potential against SARS-CoV-2 (14).

To determine whether other antibody families have broad neutralization activity, representative clones of Family A, B, D, and E antibodies (14) were evaluated in a neutralization assay (see Materials and Methods) against rcVSV-S WA-1 and rcVSV-S expressing SARS-CoV-2 variants (Fig. 1). FG-10A3 was used as a positive control for neutralization. RcVSV-S virions expressing the WT-D614G (WA-1), B.135 (Beta), B.617.2 (Delta), and BA.1 (Omicron) Spike variants were used as a proxy in order to examine the neutralization of SARS-CoV-2 Spike-mediated infection under BSL2 conditions. The rcVSV-S virions were preincubated with representative clones of Family A clone 10D6 (FA-10D6), Family B clone FB-1D10 (FB-1D10), Family D clone 2C1 (FD-2C1), Family E clone 14G5 (FE-14G5), and Family G clone 10A3 (FG-10A3) prior to infection of Vero-ACE2-TMPRSS2 cells and analyzed for GFP fluorescence by a Celigo cytometer (Fig. 1). The relative percent infection was determined using pretreatment with an isotype mAb control as 100% infection. The results revealed that FA-10D6 and FD-2C1 (Fig. 1A and C) inhibited infection of rcVSV-S WA-1 and Delta, suggesting these mAbs recognize a region conserved between the WA-1 and Delta Spike proteins. On the other hand, FB-1D10 neutralized rcVSV-S WA-1 and Beta, while FE-14G5 limited infection of rcVSV-S WA-1 and Omicron BA.1, respectively (Fig. 1B and D). Consistent with previous studies using SARS-CoV-2 Spike pseudoviruses and SARS-CoV-2 variants (14), FG-10A3 effectively neutralized rcVSV-S WA-1, Beta, Delta, and Omicron BA.1 (Fig. 1E) with EC_50_ values from 6.2 to 11 ng/mL (Table 2). The broad neutralization of FG-10A3 was not solely due to its affinity (*K*_*D*_) for SARS-CoV-2 Spike WA-1 RBD (0.13 nM) or specific length of the CDR3 region, as FA-10D6, FB-1D10, FD-2C1, and FE-14G5 demonstrated higher affinities (0.057–0.11 nM) (Table 1). Interestingly, the *K*_*D*_ of FG-10A3 was slightly higher for RBD than the S1 subunit (14) likely due to the stability and compact structure of the RBD. The capacity of FG-10A3 to broadly neutralize rcVSV-S variants was similar to a commercially engineered version of FG-10A3, denoted STI-9167 (14). As such, identifying FG-10A3’s epitope will better define the RBD region conserved across these SARS-CoV-2 variants.

**TABLE 2:** Half-maximal inhibitory concentrations (EC_50_) for anti-RBD mAbs against rcVSV-S variants

	rcVSV-S variant (EC_50_ [ng/mL])						
mAb	WA-1	Beta	Delta	Omicron (BA.1)	WA-1 + F486S	WA-1 + F486L	WA-1 + F486V
FA-10D6	33.5	>1,000	54.7	>1,000	101.8	13.5	24.4
FB-1D10	2.19	6.42	>1,000	>1,000	>1,000	>1,000	>1,000
FD-2C1	6.2	>1,000	173.4	>1,000	110.2	2.45	2.84
FE-14G5	72.7	>1,000	>1,000	26.4	75.9	16.8	12.7
FG-10A3	6.21	11	9.31	10.8	>1,000	150.1	102.6

We next evaluated the inhibitory function of the anti-RBD mAbs using an ACE2 binding assay. Recombinant SARS-CoV-2 (WA-1) RBD-Fc preincubated with FA-10D6, FB-1D10, FD-2C1, FE-14G5, FG-10A3, or non-RBD control antibody were added to HEK-293 cells expressing ACE2, followed by flow cytometry analysis for RBD-Fc binding to ACE2 (Fig. 1F). The binding of RBD-Fc to ACE2-expressing cells yielded a strong fluorescent signal that was dramatically reduced upon preincubation with anti-RBD mAbs. These data indicate that the anti-RBD mAbs limited SARS-CoV-2 infection by preventing Spike binding to ACE2.

### Generation of resistant rcVSV-S WA-1 virions against the anti-RBD mAb FG-10A3

Based on the neutralization profile of FG-10A3 against SARS-CoV-2 variants, we planned to identify the key residues of the Spike protein targeted by FG-10A3 by generating antibody-resistant rcVSV-S (WA-1) virions (33). The amplification of rcVSV-S in the presence of the mAb would provide a selective context for the identification of antibody-resistant viruses elicited over several passages. To that end, we utilized rcVSV-S WA-1 to select for mutants resistant to FG-10A3 (Fig. 2). Briefly, rcVSV-S WA-1-infected Vero-E6 cells were incubated with FG-10A3 (0–3,000 ng/mL) for 3 days followed by analysis of GFP-positive cells. Supernatant from the well with the highest mAb concentration that revealed a >50% infection was selected for additional rounds of incubation with the range of FG-10A3 concentrations (Fig. 2A). Using this strategy, resistant rcVSV-S WA-1 was observed at passage 6, and we continued to collect viruses from an additional two passages. To validate the generation of antibody-resistant rcVSV-S WA-1, we evaluated the neutralization capacity of FG-10A3 against virions collected from all passages (Fig. 2B). FG-10A3 neutralized the virus harvested from rcVSV-S WA-1 passages 1–5 with EC_50_ values comparable to the parent rcVSV-S WA-1 strain. However, FG-10A3 was unable to inhibit the infection of the virus collected from passages 6–8, implying that the rcVSV-S WA-1 virions from these passages were insensitive to FG-10A3.

### Identification of RBD mutations that give rise to FG-10A3-resistant rcVSV-S WA-1 virions

**Fig 3:**
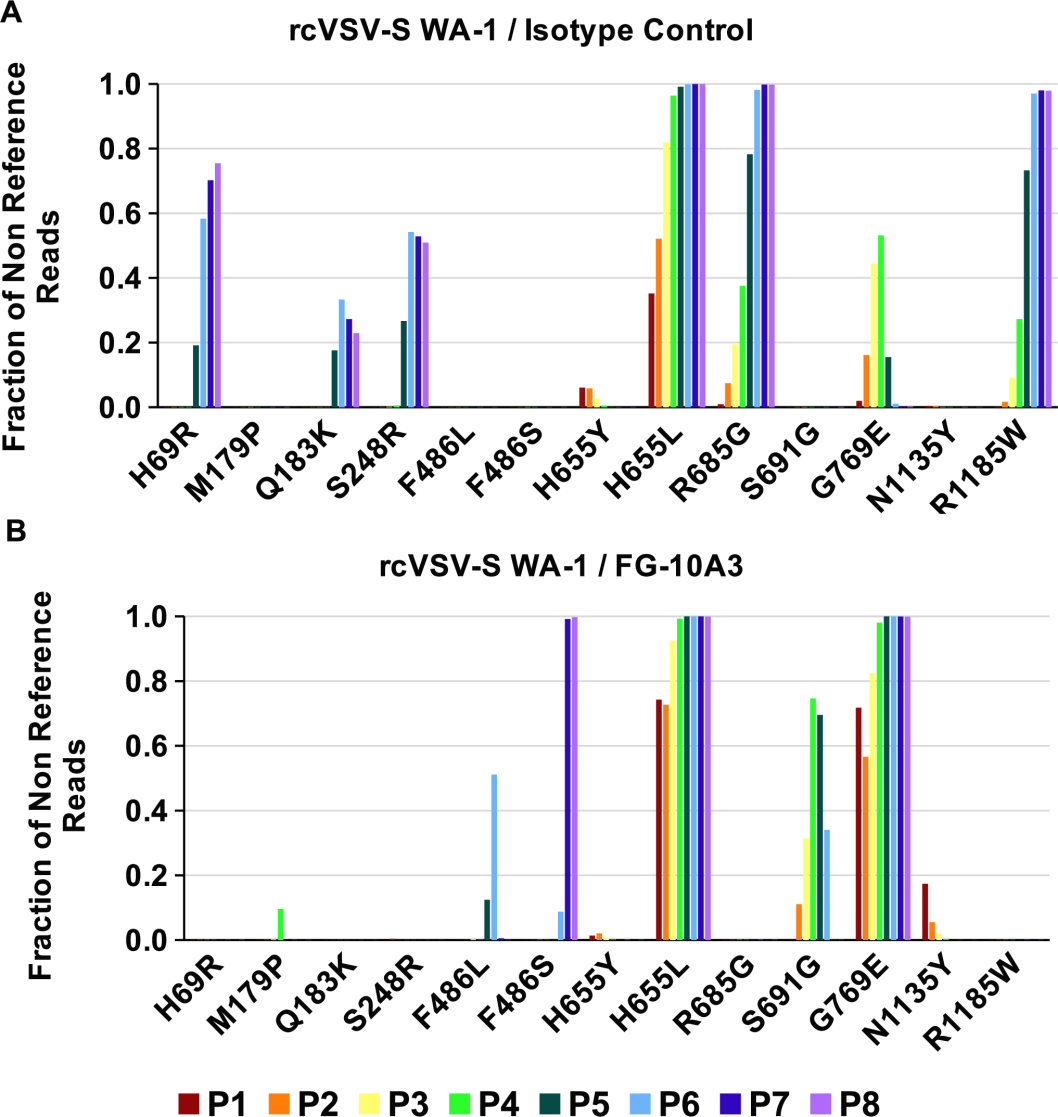
Identification of amino acid changes in rcVSV-S WA-1 virions resistant to FG-10A3. Viral RNA extracted from eight sequential passages of rcVSV-S WA-1 incubated with isotype control mAb (**A**) or with FG-10A3 (**B**) was amplified via RT-PCR and subjected to Illumina sequencing and analysis of Spike mutational variants. Changes in amino acids at different residues of the Spike protein are represented by single-letter amino acid code and quantified as a fraction of total reads compared to non-reference reads. Quantified reads from passages (P) 1–8 are distinguished by color.

Viral RNA was extracted from passages 1–8 of rcVSV-S WA-1 incubated with isotype control mAb or FG-10A3. The Spike region of rcVSV-S WA-1 virions was PCR-amplified and sequenced at a total depth of 7.7 million reads across all samples, with 4.9 million aligning to SARS-CoV-2 Spike. Samples contained between 10,700 and 75,200 aligned reads, with a median of 17,300. After variant calling, only those variants both resulting in an amino acid change and meeting a threshold of 10% of the total reads in any one sample are shown (Fig. 3). Importantly, analysis of mutations in the RBD (aa 319–541) identified changes at position F486, which were unique only to the FG-10A3-incubated rcVSV-S WA-1 virions compared to virions incubated with isotype control (Fig. 3A versus B). A mutation from F to L arose at passages 5 and 6 and was then outcompeted by a change to S at passage 6, which quickly reached >99% prevalence in reads by passage 7 (Fig. 3B). These findings imply that F486 is a key residue for FG-10A3 neutralization of SARS-COV-2. Interestingly, rcVSV-S WA-1 passaged in the presence of isotype control mAb and FG-10A3 identified additional mutations in the S1 (aa 1–685) and S2 (aa 686–1273) domains of the Spike protein (Fig. 3). Mutations H69R, Q183K, S248R, H655L, R685G, G769E, and R1185W were observed during the passage of rcVSV-S WA-1 incubated with isotype antibody (Fig. 3A), while H655L, S691G, and G769E were identified in rcVSV-S WA-1 virions passaged with FG-10A3 (Fig. 3B). These amino acid changes may have arisen in order to enhance the proliferation and dissemination of rcVSV-S WA-1 *in vitro* in Vero-E6 cells, where virus entry is dependent on cathepsin-mediated cleavage of the Spike protein. Indeed, passaging of SARS-CoV-2 in Vero-E6 cells lacking TMPRSS2 or other surface proteases can select for viruses containing mutations within the furin cleavage site, which includes residue R685 (34). Collectively, the experimental strategy of sequencing mAb-resistant virus strains generated *in vitro* can identify residues of the target viral protein which are critical for antibody binding.

### A polar residue at Spike position 486 dramatically reduces the neutralization capacity of FG-10A3

**Fig 4:**
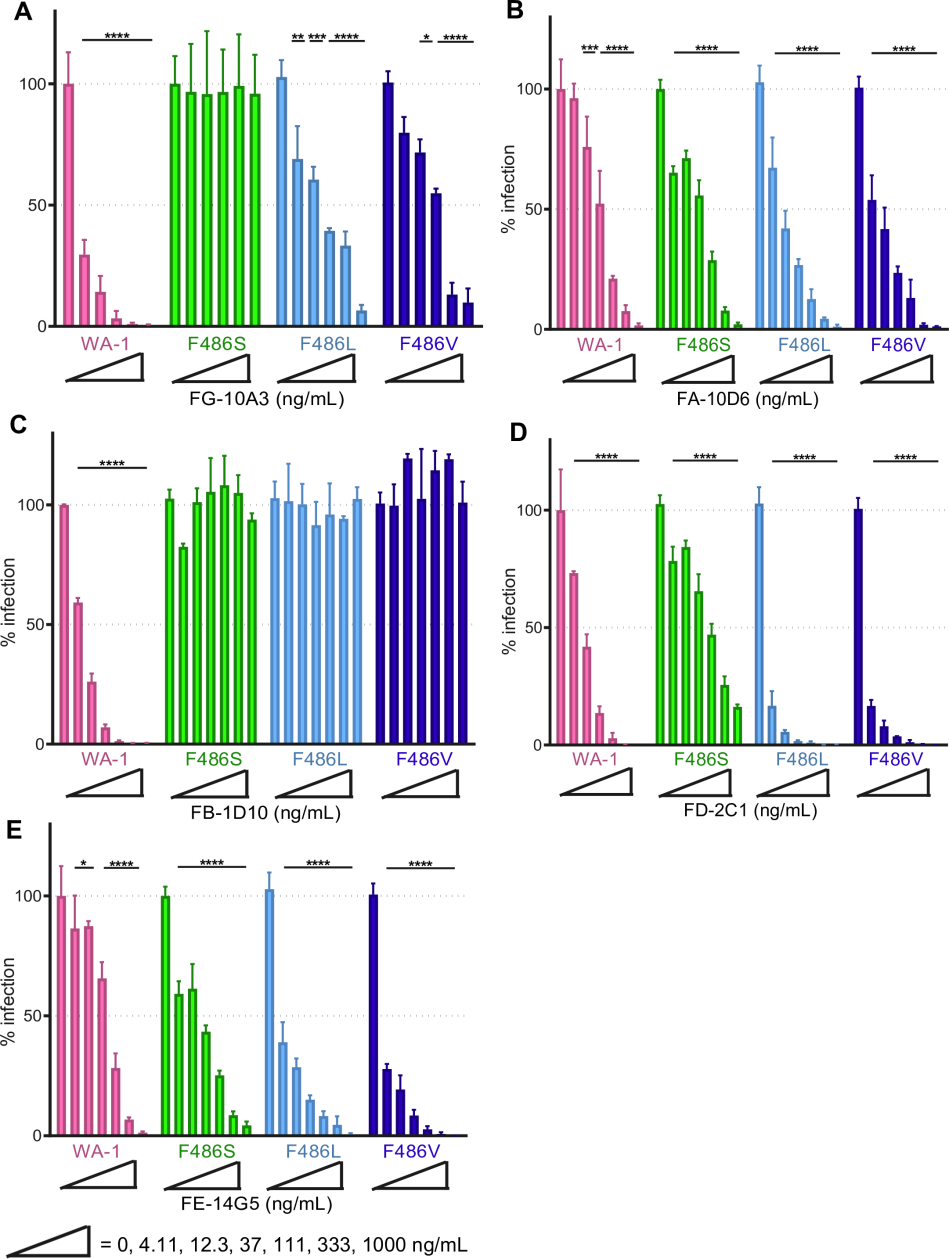
Analysis of anti-SARS-CoV-2 mAbs against rcVSV-S F486 point mutants. FG-10A3 (**A**), FA-10D6 (**B**), FB-1D10 (**C**), FD-2C1 (**D**), and FE-14G5 (**E**) mAbs (0–675 ng/mL) were evaluated with a neutralization assay against rcVSV-S WA-1 and rcVSV-S WA-1 containing point mutants F486S, F486L, and F486V. Infection was analyzed using the neutralization assay. Error bars represent the standard deviation from the mean of three samples (serving as technical replicates). Data depicted are that of a single representative experiment out of two independent experimental replicates performed. Statistical significance is denoted as follows: *P < 0.05; **P < 0.01; ***P < 0.001; and ****P < 0.0001.

To validate the importance of F486 in FG-10A3-mediated neutralization, we performed neutralization assays using rcVSV-S WA-1 variants in which the F486 was modified to a leucine (rcVSV-S F486L), valine (rcVSV-S F486V), or serine (rcVSV-S F486S) (Fig. 4A). rcVSV-S F486S was entirely resistant to neutralization by FG-10A3 and its commercial counterpart STI-1967 (Fig. S1), supporting the generation of FG-10A3-resistant rcVSV-S WA-1 virions (Fig. 2 and 3). Interestingly, neutralization of rcVSV-S F486L and rcVSV-S F486V by FG-10A3 was only somewhat reduced compared to the parent rcVSV-S WA-1, with EC_50_ values of 150.1 and 102.6 ng/mL, respectively (Table 2). These results suggest that the substitution of a phenylalanine to nonpolar residues leucine or valine only slightly impacted mAb binding to Spike while still permitting antibody-mediated neutralization. Thus, the conservation of the hydrophobic nature of residue 486 within the Spike protein is important to the Spike/FG-10A3 interaction.

Previous nucleotide sequence analysis performed on the anti-RBD mAbs (14) demonstrated that our panel of mAbs are genetically distinct; this combined with their variable neutralization efficacies against distinct SARS-CoV-2 Spike variants implies that the mAbs are highly likely to recognize and bind to different epitopes on the SARS-CoV-2 Spike. As such, mutations within Spike at position F486 may not impact the neutralization capacity of other anti-RBD mAbs. To address this, we evaluated the neutralization capacity of FA-10D6, FB-1D10, FD-2C1, and FE-14G5 against rcVSV-S WA-1 carrying F486 point mutants (Fig. 4B through F). RcVSV-S WA-1, F486S, F486L, and F486V were subjected to the neutralization assay. FA-10D6, FD-2C1, and FE-14G5 limited infection of all F486 point mutants as determined by EC_50_ values (Table 1) implying these mAbs target a different epitope within RBD than that bound by FG-10A3. FB-1D10 was unable to neutralize any of the F486 point mutants suggesting that F486 is important for mAb recognition. These data indicate that the RBD-targeting mAbs have diverse epitopes and that the F486S mutation does not dramatically impact the overall structure of Spike.

### Cryo-EM structure of STI-9167 with SARS-CoV-2 Spike

**Fig 5:**
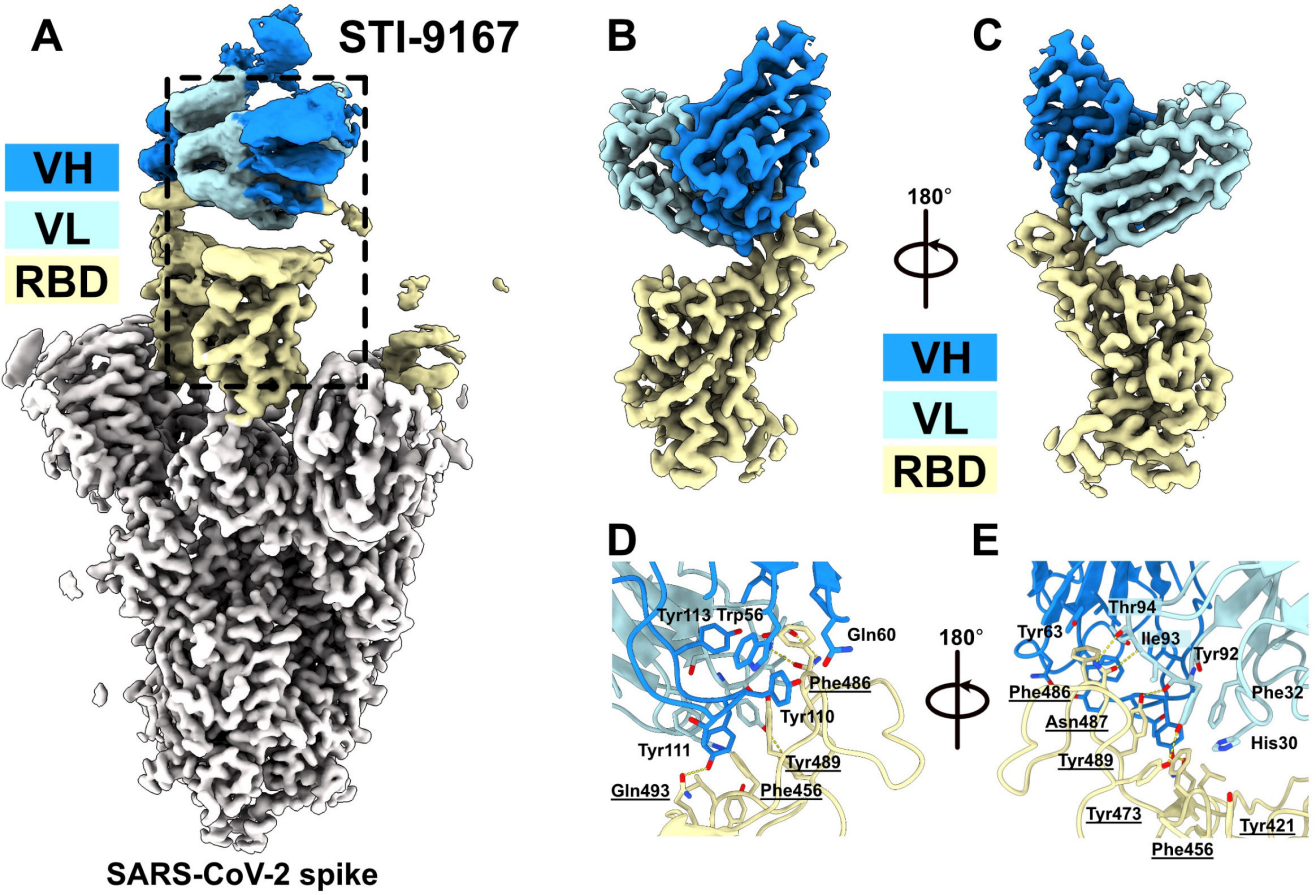
Structural analysis of anti-Spike RBD mAb STI-9167 with SARS-CoV-2 Spike. Global (**A**) and local (**B and C**) cryo-EM reconstructions of the STI-9167 Fab in complex with SARS-CoV-2 Spike are shown with RBD colored in yellow, non-RBD regions in gray, and Fab variable heavy and light chains in dark and light blue, respectively. Two 180° rotated views (**D and E**) display details of the intermolecular interactions, with the most prominent interacting residues annotated. RBD-interacting residues are both numerically notated and underlined.

To define the mAb epitope and the intermolecular interactions occurring at the antibody:antigen interface, we determined the structure of STI-9167 Fab (a therapeutically modified version of FG-10A3; see reference 14) in complex with SARS-CoV-2 Spike using single-particle cryo-EM (Fig. 5; Fig. S2; Table S1). The global structure indicated that STI-9167 binds to Spike along its threefold axis and engages the RBD in the “up” configuration (Fig. 5A). We next performed local refinement of the STI-9167 Fab/RBD complex to resolve the amino acid side-chain contacts at the interface. The locally refined map, at 3.16 Å nominal resolution, indicated that the Fab contacts the disulfide-stabilized 470–490 loop with both its variable heavy and light chains (Fig. 5B through E). The buried interaction surface area of 610 Å^2^ is relatively small compared to other anti-RBD mAbs, such as bamlanivimab, which has an interaction surface area of 836 Å^2^.

At the nexus of the interactions within the antibody:antigen interface is the RBD residue F486, which protrudes into a hydrophobic cleft formed by aromatic residues supplied predominantly by the HCDR2 and HCDR3 antibody loops. Indeed, the antibody residues W56, Y63, Y111, and Y113 wrap around F486, bolstering our findings with the rcVSV-S F486 point mutants. In addition, most polar contacts within this interface involve the Fab light chain, which forms hydrogen bonds with RBD Y473, N487, and Y489. Interestingly, the majority of polar contacts involve the main chain carbonyls supplied by the mAb LCDR3 Y92, I93, and T94 residues (Fig. 5E). In summary, the small binding interface and main-chain contacts likely contribute to the broadly reactive and potently neutralizing activity of mAb FG-10A3/STI-9167 against the tested SARS-CoV-2 VoCs.

### Analysis of FG-10A3 neutralization of emergent Spike VoCs

**Fig 6:**
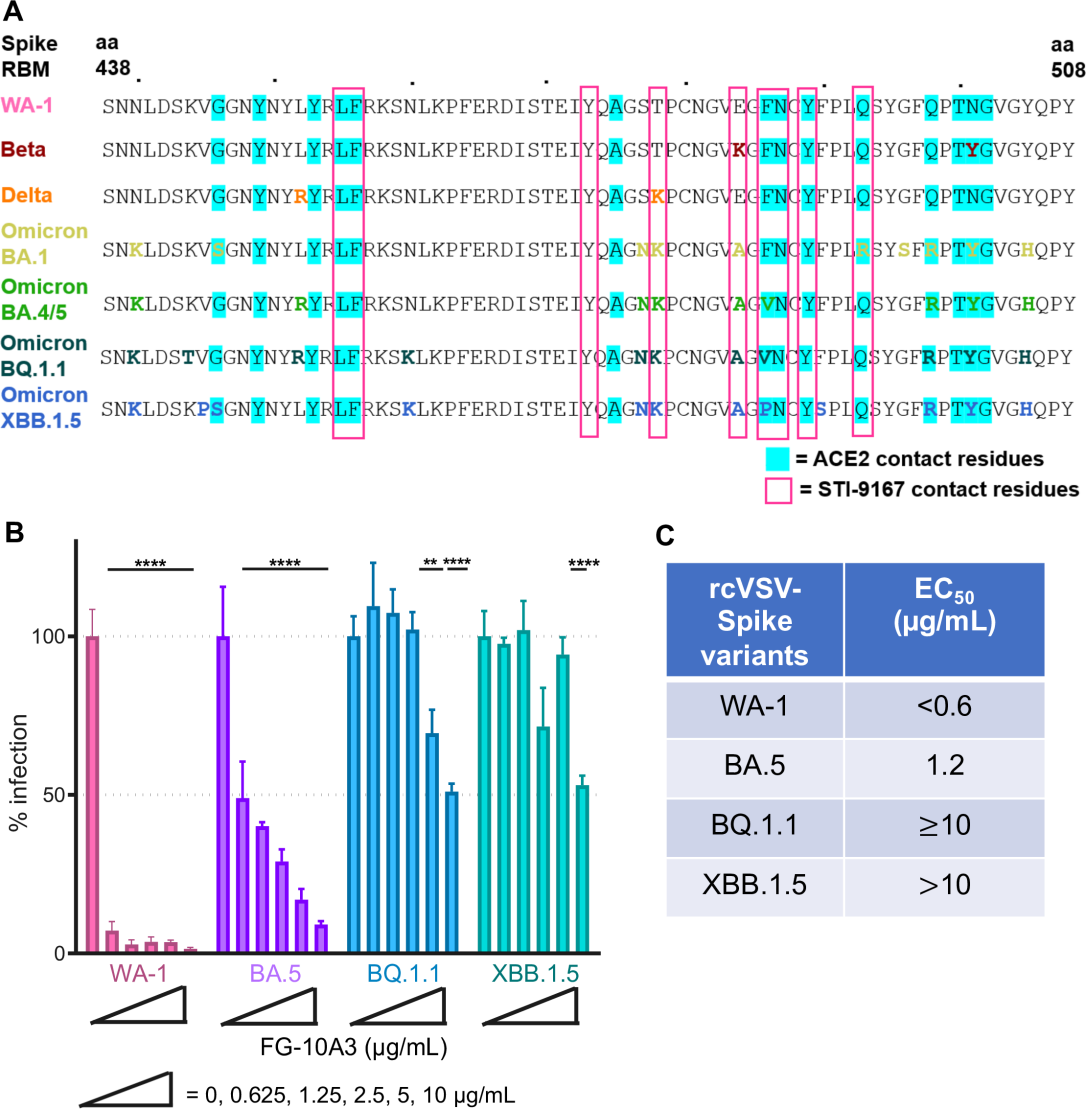
Neutralization of FG-10A3 against SARS-CoV-2 variants. (**A**) The ACE2 (cyan highlights) and FG-10A3 derivative STI-9167 (pink boxes) contact residues of the Spike receptor-binding motif (aa 438–508) of SARS-CoV-2 WA-1, Beta, Delta, and the Omicron sublineages BA.1, BA.5, XBB.1.5, and BQ.1.1 are shown. The amino acid sequence of the RBD is represented using the single-letter amino acid code. (**B**) FG-10A3 was evaluated with a neutralization assay against rcVSV-S WA-1 (WA-1) and rcVSV-S expressing Spike variants Omicron BA.5, Omicron XBB.1.5, and Omicron BQ.1.1 preincubated with mAb (0–10 μg/mL) followed by infection of Vero-Ace2/TMPRSS2 cells and analyzed using the neutralization assay. Error bars represent the standard deviation from the mean of three samples (serving as technical replicates). Data depicted are that of a representative experiment out of two independent experimental replicates performed. (**C**) The EC_50_ values (μg/mL) determined from the FG-10A3 neutralization assay for rcVSV-S WA-1, BA.5, BQ.1.1, and XBB.1.5. Statistical significance is denoted as follows: **P < 0.01 and ****P < 0.0001.

The cryo-EM-defined epitope of FG-10A3 overlaps with numerous ACE2-binding residues of the Spike protein (Fig. 6A). Our data suggest that a hydrophobic residue at position 486 plays an important role in FG-10A3’s neutralization activity based on the neutralization profile of rcVSV-S F486L and F486V and the cryo-EM image of STI-9167/Spike (Fig. 4 and 5). To further explore this point, we evaluated the effectiveness of FG-10A3 against rcVSV-S virions expressing Spike from currently circulating SARS-CoV-2 variants which contain naturally acquired modifications at position 486 and elsewhere in the Spike protein. We generated rcVSV-S particles pseudotyped with SARS-CoV-2 Spike variants BA.5, BQ.1.1, and XBB.1.5 because they contain a valine or proline at position 486 (Fig. 6A), as >95% of circulating variants contain one of these two residues at position 486 as of July 2023 (35). We excluded the analysis of variants containing S486, as these variants are expected to be completely insensitive to FG-10A3. Neutralization assays were performed with FG-10A3 (0–10 µg/mL) against rcVSV-S WA-1, BA.5, XBB.1.5, and BQ.1.1 (Fig. 6B). As expected, FG-10A3 limited infection of rcVSV-S WA-1 (EC_50_: <0.010 µg/mL). Comparatively, FG-10A3 was less effective at neutralizing rcVSV-S BA.5 and rcVSV-S BQ.1.1 with EC_50_ values of 1.2 and ≥10 µg/mL (Fig. 6C), respectively. The decrease in the neutralization of rcVSV-S BA.5 and rcVSV-S BQ.1.1 was striking, as the neutralization studies performed with FG-10A3 against rcVSV-S WA-1(F486V) were similar to rcVSV-S WA-1 (Fig. 4A; Table 2). These results imply that additional mutations within the Spike protein may influence epitope exposure and mAb binding to Spike. For example, the BQ.1.1 variant contains a mutation at position 460 from asparagine to lysine (N460K), as does XBB.1.5. This change from a nonpolar to positively charged amino acid, though not occurring at a residue that makes direct contact with FG-10A3/STI-9167, may nonetheless impact the exposure of other mAb contact residues or the molecular structural elements necessary for mAb recognition and binding to the Spike RBM. Mutations within Spike that lie outside its receptor-binding domain may have indirect effects with regards to abrogating antibody-mediated neutralization; for example, a mutation in the Spike N-terminal domain (NTD) at position 252 from glycine to valine is critical for enhancing XBB.1.5’s evasion of humoral immunity, as this residue is frequently targeted by antibodies specific for the NTD (36).

Notably, the mutation of residue 486 to proline in rcVSV-S XBB.1.5 significantly reduced the ability of FG-10A3 to limit infection (Fig. 6B and C; EC_50_ > 10 mg/mL). This supports the overarching paradigm that the loss of hydrophobicity within residue 486 of Spike provides virus escape from mAb neutralization. Collectively, our findings demonstrate the limitations of FG-10A3-mediated neutralization through the modification of key contact residues.

## DISCUSSION

A novel coronavirus was identified in December 2019 as the causative agent of the disease referred to as COVID-19 (37). In the intervening time since its isolation, the coronavirus SARS-CoV-2 has spread to virtually every part of the globe, while also dramatically expanding in regard to its catalog of circulating “variant” strains, especially those containing mutational variation within viral proteins such as Spike. Defining and evaluating such mutations is important for predicting and assessing the continued efficacy of existing antiviral therapeutics and vaccines. In this work, we have utilized a combinatorial approach to identify the epitope of the broadly reactive anti-RBD mAb FG-10A3/STI-9167. A residue within the RBD which is critical for FG-10A3 neutralizing capacity was uncovered by experimentally generating FG-10A3-resistant rcVSV-S WA-1 virions (Fig. 2 to 4), in conjunction with cryo-EM of the STI-9167/Spike complex (Fig. 5) and rcVSV-S variant neutralization assays. Together, these data support the importance of residue F486 in the Spike protein for mediation of neutralization by FG-10A3/STI-9167. The experimental pipeline utilized to identify the epitope of a broadly neutralizing anti-SARS-CoV-2 mAb highlights a powerful *in vitro* strategy to define the therapeutic potential of biologics for current and future viral variants.

At the time of initial characterization of these antibodies, all SARS-CoV-2 VoCs were sensitive to FG-10A3/STI-9167 due to an overall lack of mutational changes within Spike RBD residues that make contact with the antibody. However, some SARS-CoV-2 subvariant lineages that have more recently emerged contain changes at position 486, namely, BA.5 and BQ.1.1 (V486) as well as XBB.1.5 (P486) (Fig. 6A). Indeed, non-hydrophobic residues serine and proline at position 486 limit neutralizing capability of mAbs against circulating SARS-CoV-2 variants (38) and laboratory-generated mutants (39). Moreover, FG-10A3’s ability to recognize and bind to its Spike epitope was further impacted by changes in residues beyond the antibody’s contact residues. In our neutralization assays with rcVSV-S BA.5 and rcVSV-S BQ.1.1 (Fig. 6B and C), we observed a 10- and 100-fold loss in neutralization activity when compared to rcVSV-S WA-1 F486V point mutant (Fig. 4A; Table 2). This loss in neutralization activity may be explained in part by the presence of other amino acid changes within the Spike RBD of Omicron BA.5 and BQ.1.1 outside of the antibody contact residues that can influence the structure and stability of Spike. As such, examination of individual and combinatorial effects of changes within the SARS-CoV-2 Spike protein on immune evasion merits further detailed investigation, especially as new variants continue to emerge.

Importantly, serine at position 486 may incur a fitness “penalty” for the virus, in the form of impaired binding to the ACE2 cellular receptor (40). The structure of SARS-CoV-2 Spike RBD in association with ACE2 supports the idea that the presence of a large, hydrophobic residue at Spike position 486 facilitates efficient receptor binding via engagement with a hydrophobic “pocket” made by ACE2 residues L79, M82, and Y83 (41). This perhaps contributes toward explaining why S486 is only found in <1% of circulating variants worldwide as of August 2023 (35), whereas ~98% of current circulating variants contain a proline at residue 486, a substitution that likely maintains a favorable RBD:ACE2 interaction. Interestingly, the prevalence of S486 in variants has decreased by tenfold since early 2023 when it was found in ~9% of sampled circulating variants. At the same time, P486 in variants had a similar relative prevalence of ~11%, with the majority of variants (~74%) containing valine at position 486 (35). Thus, consideration of the totality of the evolutionary “landscape,” as well as changes within that landscape over time, is critical when assessing the viability and relevance of SARS-CoV-2 variations in the context of *in vivo* pathogenesis. By contrast, the relatively permissive fitness environment established by our *in vitro* rcVSV-S reporter system permits a broader, more enhanced identification of potential limitations to a therapeutic’s efficacy that may lie outside of the rigors of selection imposed by the pre-existing evolutionary environment.

Knowledge of the mAb’s epitope and its molecular underpinnings allows us to preemptively predict the location and nature of mutational variants in Spike that would limit antibody neutralization. This more broadly highlights the value of experimentally soliciting viral escape mutants to neutralizing antibodies, not only to characterize those mAbs’ epitopes but also as a means for possibly predicting mutations that may arise as SARS-CoV-2 variants continue to proliferate under dynamically changing conditions of evolutionary fitness. Indeed, neutralization assays to determine FG-10A3’s activity against rcVSV-S (WA-1) F486 point mutants and currently circulating Spike VoCs highlight the importance of antibody contact residues as well as residues which lay outside the epitope.

The discovery and production of human mAbs in transgenic animals have therapeutic advantages such as permitting *in vivo* affinity maturation, clonal selection for subsequent antibody optimization, and generation of mAbs targeting diverse VoCs prior to their widespread circulation (42). This work underscores the utility of transgenic mice and the potential of an immunization strategy with a viral protein domain as a means of generating a diverse panel of mAbs that target specific antigens. More broadly, our work highlights the use of complementary biological and biochemical strategies including BSL2 rcVSV neutralization assays, elicitation of antibody-resistant viruses, and cryo-EM-based structural analysis of antibody:antigen complexes to define the therapeutic breadth of an antibody biologic.

## Data Availability

Requests for material should be addressed to Domenico Tortorella (domencio.tortorella@mssm.edu) and Goran Bajic (goran.bajic@mssm.edu). The EM maps have been deposited in the Electron Microscopy Data Bank (EMDB) under accession code EMD-28537 and the accompanying atomic coordinates in the Protein Data Bank (PDB) under accession code 8EQF. The aligned micrographs are available on the Electron Microscopy Public Image Archive (EMPIAR) under accession number EMPIAR-11341.

## SUPPLEMENTAL MATERIAL

Fig. S1 mbio.02477-23-s0001.eps Neutralization of STI-9167 against rcVSV-S variants.

Fig. S2 mbio.02477-23-s0002.eps Cryo-EM data processing scheme and local resolution estimation of the final map.

Table S1 mbio.02477-23-s0003.docx Data from the cryo-EM study.

ASM does not own the copyrights to Supplemental Material that may be linked to, or accessed through, an article. The authors have granted ASM a non-exclusive, world-wide license to publish the Supplemental Material files. Please contact the corresponding author directly for reuse.
