# Supplementary material for: An in vitro experimental pipeline to characterize the epitope of a SARS-CoV-2 neutralizing antibody
Source: mBio. 2023 Dec 6;15(1):e02477-23. doi: 10.1128/mbio.02477-23 (PMC10870823; doi:10.1128/mbio.02477-23)
Supplement: Table S1 — Data from the cryo-EM study. [file mbio.02477-23-s0003.docx]

**Supplementary Materials**

| **Table S1: Cryo-EM data collection and model validation statistics** | |
| --- | --- |
| **Data Collection** | |
| **Grid type** | UltrAuFoil gold R0.6/1 |
| **Microscope/voltage/detector** | Totan Krios/300 kV/Gatan K3 summit |
| **Magnification** | 105,000 |
| **Recording mode** | counting |
| **Total dose** | 58.92 e-/Å^2^/s |
| **Pixel size** | 0.826 Å/pixel |
| **Defocus range** | –1.5 to –0.5 μm |
| **No. micrographs used** | 20,590 |
| **Total particles picked** | 1,410,814 |
| **Model Validation** | |
| **Composition (#)** |  |
| **Chains** | 4 |
| **Atoms** | 3348 |
| **Residues** | Protein: 422 Nucleotide: 0 |
| **Ligands** | NAG: 2 |
| **Bonds (RMSD)** | |
| **Length (Å) (# > 4sigma)** | 0.005 (0) |
| **Angles (°) (# > 4sigma)** | 0.562 (0) |
| **MolProbity score** | 1.70 |
| **Clash score** | 5.82 |
| **Ramachandran plot (%)** | |
| **Outliers** | 0.00 |
| **Allowed** | 5.53 |
| **Favored** | 94.47 |
| **Rotamer outliers (%)** | 0.00 |
| **Cbeta outliers (%)** | 0.00 |
| **Peptide plane (%)** | |
| **Cis proline/general** | 10.0/0.0 |
| **Twisted proline/general** | 0.0/0.0 |
| **CaBLAM outliers (%)** | 2.20 |
| **ADP (B-factors) min/max/mean** | |
| **Protein** | 5.25/106.21/63.35 |
| **Ligand** | 53.49/73.42/64.79 |
| **Data** | |
| **Lengths (Å)** | 56.17, 66.08, 109.03 |
| **Angles (°)** | 90.00, 90.00, 90.00 |
| **Supplied Resolution (Å)** | 3.2 |
| **Resolution Estimates (Å)** | Masked Unmasked |
| **d FSC (half maps; 0.143)** | 3.1 3.2 |
| **d 99 (full/half1/half2)** | 2.3/1.9/1.9 2.3/1.8/1.8 |
| **d model** | 2.2 2.2 |
| **d FSC model (0/0.143/0.5)** | 1.7/1.9/3.2 1.7/1.9/3.3 |
| **Map min/max/mean** | 0.00/1.75/0.02 |
| **Model vs. Data** | |
| **CC (mask)** | 0.79 |
| **CC (box)** | 0.73 |
| **CC (peaks)** | 0.67 |
| **CC (volume)** | 0.80 |
| **Mean CC for ligands** | 0.70 |
